# Direct Electricity Production from *Nematostella* and *Arthemia*’s Eggs in a Bio-Electrochemical Cell

**DOI:** 10.3390/ijms232315001

**Published:** 2022-11-30

**Authors:** Yaniv Shlosberg, Vera Brekhman, Tamar Lotan, Lior Sepunaru

**Affiliations:** 1 Department of Chemistry and Biochemistry, University of California at Santa Barbara, Santa Barbara, CA 93106, USA; 2 Marine Biology Department, Leon H. Charney School of Marine Sciences, University of Haifa, Haifa 3498838, Israel

**Keywords:** *Nematostella*, *Artemia*, sea anemones, electrochemical cell, bio electrochemical cell, NADH, fluorescence, Cytochrome C, clean energy

## Abstract

In recent years, extensive efforts have been made to develop clean energy technologies to replace fossil fuels to assist the struggle against climate change. One approach is to exploit the ability of bacteria and photosynthetic organisms to conduct external electron transport for electricity production in bio-electrochemical cells. In this work, we first show that the sea anemones *Nematostella vectensis* and eggs of *Artemia* (brine shrimp) secrete redox-active molecules that can reduce the electron acceptor Cytochrome C. We applied 2D fluorescence spectroscopy and identified NADH or NADPH as secreted species. Finally, we broaden the scope of living organisms that can be integrated with a bio-electrochemical cell to the sea anemones group, showing for the first time that *Nematostella* and eggs of *Artemia* can produce electrical current when integrated into a bio-electrochemical cell.

## 1. Introduction

The increasing concern caused by climate change inspired scientists to develop new eco-friendly energy solutions to replace existing technologies that emit CO_2_ into the atmosphere. One of the approaches is to exploit living organisms as an energy source capable of catalyzing redox reactions in bio-electrochemical cells. This approach was first applied a few decades ago in microbial fuel cells (MFCs) [1]. In this method, exo-electrogenic bacteria can serve as electron donors at the anode of a bio-electrochemical cell (BEC). The electron source in MFCs originates from the mitochondrial biochemical activity of the cells [2]. External electron transport can be conducted by mediated electron transport [3,4,5,6,7,8] or direct electron transport [9,10,11,12]. In the case of mediated electron transport, the electrons are transferred by redox mediators such as quinone and phenazine derivatives [3,4,5,6,7,8]. In the case of direct electron transport, the electrons are directly shuttled between the cells and the anodes via *Pili* [13,14,15,16] or metal respiratory complexes [17,18]. In addition to bacteria, yeast was also reported to produce electricity in BECs, showing that the ability to perform external electron transport may exist in various microorganisms and is not restricted to bacteria [19]. The utilization of isolated components benefits from a more concentrated active material for electricity generation [20,21]; however, unlike whole cells, they lack repair mechanisms and may be less stable [22]. Additionally, the cost of the purification process may be a significant economic consideration while trying to elaborate such methods from a basic concept into actual applicative technologies. An interesting approach is to combine the whole plants with MFCs. In this method, the natural photosynthesis process is exploited to produce organic material [23]. The organic material is released from the plant’s root, which later feeds the bacteria as a continuous energy source that produces electricity in an MFC architecture [24,25,26]. Recently, it was reported that bio-photo electrochemical cells (BPECs) are not limited to micro-organisms and can be based on photosynthetic macroorganisms such as seaweeds [27] and aqueous and terrestrial plants [28] as electron donors. Interestingly, macroorganisms-based BPECs have produced an electrical current in both dark and light that was about 100–1000 times greater than photosynthetic microorganisms [29]. Rather than using whole microorganisms in BECs, electrical current can be produced via photon excitation by utilization of isolated photosynthetic components in different levels of purification, such as Thylakoid membranes [22], Chloroplasts [30], and Photosystems [31,32,33,34,35,36,37]. Another approach is the utilization of isolated enzymes/non-photosynthetic components that can catalyze electrochemical reactions in BECs [38,39].

To date, BECs are limited solely to the utilization of microorganisms, photosynthetic macroorganisms, and isolated photosynthetic components. In this work, we present for the first time a BEC construct based on redox-active donors extravasated from species belonging to the sea anemones group. We show that *Nematostella vectensis* and *Artemia*’s eggs can secrete molecules that reduce Cytochrome C (Cyt C). Using 2D-fluorescence spectroscopy, we identified that the redox-active molecules NADH and NADPH (NAD(P)H) are among these molecules.

## 2. Results and Discussion

### 2.1. Artemia’s Eggs and Nematostella Secrete NAD(P)H to the External Solution

**Figure 1:**
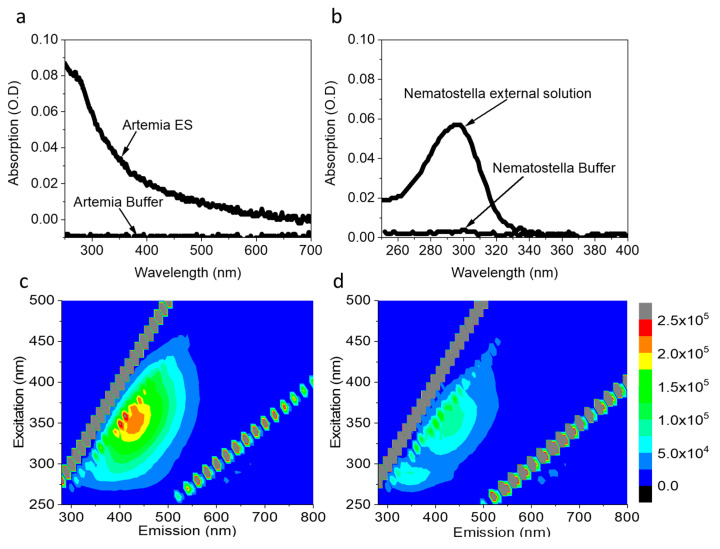
*Artemia*’s eggs and *Nematostella* release molecules to the external solution, including NAD(P)H. (**a**) Absorption spectra of the pure buffer of *Artemia* and its external solution. (**b**) Absorption spectra of the pure buffer of *Nematostella* and its external solution. (**c**) 2D-fluorescence spectra of the external solution of *Artemia*. (**d**) 2D-fluorescence spectra of the external solution of *Nematostella*. The lines of diagonal spots in all of the maps presented here and in the following figures result from the light scattering of the Xenon lamp and Raman scattering of the water [43].

Previous studies show that aquatic microorganisms and seaweeds can secrete redox-active molecules, including NAD(P)H [27,40,41]. Such a release of redox-active molecules can be utilized to produce electricity in a bio-electrochemical cell. We wished to explore whether organisms from the sea anemones group such as *Nematostella* or biocomponents such as *Artemia* eggs can also secrete redox-active molecules. *Artemia* eggs and *Nematostella* were chosen because of their small sizes which is optimal for an electrochemical analytic small system such as screen-printed electrodes. As a first step to explore the secretion of molecules by *Nematostella* and *Artemia* eggs into their aquatic environment, ~5 mg of *Artemia* eggs or 1 *Nematostella* unit (~75 mg) (that are sufficient to cover most or all of the area of standard electrode surface), were incubated in filtrated cultivation buffers for 1 h. Following the incubation, the organisms were gently removed using a pipette. The absorption and 2D-fluorescence spectra of the remaining solution were measured (Figure 1). The absorption spectra of *Artemia*’s eggs and *Nematostella* show a maximum around 290nm, which is dominated by the peptide backbone of biological moieties in the solution (Figure 1a,b; red curves). No significant absorption was observed for the filtrated buffers (Figure 1a,b; black curves), indicating that the peaks originated from the release of components from the *Artemia* eggs and *Nematostella*.

To gain further details on the samples’ constituents, we turned to a fluorescence study, which is more sensitive in its limit of detection. The 2D-fluorescence spectra of the external solution of the *Artemia*’s eggs showed peaks with maxima around λ_Ex_ = 280 nm, λ_Em_ = 350 nm λ_Ex_ = 350 nm, λ_Em_ = 450 nm (Figure 1c). A similar spectroscopic signature was reported for the amino acids tyrosine and tryptophan [42] and freely diffusing NAD(P)H [40]. No peaks were obtained in the buffers of *Artemia*’s eggs (Figure S1a) and *Nematostella* (Figure S1b). To evaluate the NAD(P)H concentrations, a calibration curve was prepared based on fluorescence intensities at (λ_Ex_ = 350 nm, λ_Em_ = 450 nm) (Figure S2) of increasing NADH concentrations. Based on this curve, the NAD(P)H concentrations in the external solution of the *Artemia* eggs and *Nematostella* were determined to be 1.72 µM ± 0.05 and 0.82 ± 0.08 µM, respectively.

### 2.2. Artemia’s Eggs and Nematostella Secrete Components That Can Reduce Cytochrome C

**Figure 2:**
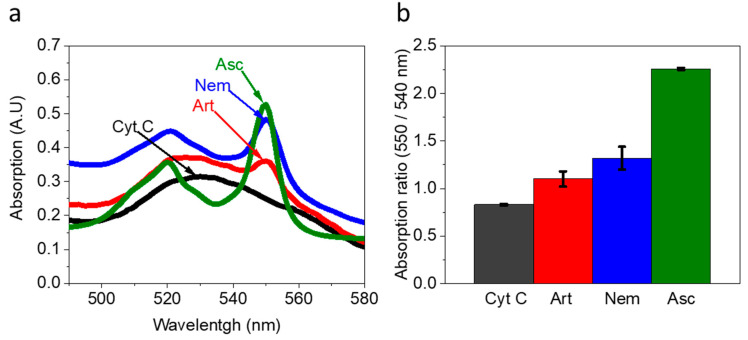
*Artemia*’s eggs and *Nematostella* secrete components that reduce Cyt C. *Artemia*’s eggs, and a *Nematostella* unit were incubated in a pure buffer for 1 h. The external solution (without the organisms) was incubated with 30 µM Cyt C for 1 h. (**a**) Absorption spectra of Cyt C (black) and Cyt C exposed to the external solution of *Artemia*’s eggs (Art, red), Cyt C exposed to the external solution of *Nematostella* (Nem, blue), and Cyt C fully reduced by Ascorbic Acid (Asc, green). (**b**) Absorption ratio 550/540 nm of Cyt C (black) and Cyt C exposed to the external solution of *Artemia*’s eggs (Art, red), Cyt C exposed to the external solution of *Nematostella* (Nem, blue), and Cyt C fully reduced by Ascorbic Acid (Asc, green). The error bars represent the standard deviation over three independent repetitions.

Following the observation that *Artemia* eggs and *Nematostella* secrete NAD(P)H and possible additional reducing molecules into their external solution, we next assessed their ability to reduce an electron acceptor in a solution, which is detrimental to electricity production via electrochemical redox reactions. For this purpose, *Artemia*’s eggs and *Nematostella* were incubated for 1 h in a fresh filtrated cultivation buffer (see materials and methods). The organisms were removed, and their external solution was added by a pipette to a Cyt C solution (30 µM, pH = 6.8) for 1 h incubation (Figure 2 and Figure S3). Incubation of Cyt C with 1 mM Ascorbic acid for 1 h, was conducted to study the absorption spectra of the fully reduced Cyt C. The advantage of using Cyt C as an electron acceptor derives from the possibility of analysing its reduction based on spectrophotometric measurements [44].

The initial solution of Cyt C before exposure to the sea animals’ external solution contained Cyt C in the oxidized form. This is evident from the classical broad peak with a maximum around 530 nm of Cyt C with Fe^3+^ ion center [45] (Figure 2a,b black curves). After exposure to the external solution of *Artemia*’s eggs and *Nematostella*, the absorption spectra exhibit two peaks with maxima around 520 nm and 550 nm, which are characteristics of Cyt C in the reduced form (Figure 2a). A similar result is observed when NADH is incubated in a solution with Cyt C (Figure S4). The absorption ratio of the fully oxidized Cyt C and the fully reduce Cyt C (after incubation with Ascorbic acid) were used to make a calibration curve for evaluating the extent of Cyt C reduction (550/540 nm) (Figure S5). The concentration of the reduced Cyt C by the external solution of *Artemia*’s eggs and *Nematostella* was determined to be about 5.6 and 10.3 µM, respectively. It is possible that by using a pipette to place the Nematostella on top of the electrodes, we may make minor damage to the cells making them spill their inner content that consists of reducing molecules. However, such a scenario may only exist for a very small amount of cells. Therefore, we do not expect it to make a significant change in the current production.

Overall, the results indicate that the secreted molecules from the sea animals exhibit redox reactions that are essential for BECs. The complementary data from the 2-D fluorescence and absorption of Cyt C indicate that NAD(P)H exists in external solution at micromolar concentrations.

### 2.3. Artemia’s Eggs Produce an Electric Current in BEC

**Figure 3:**
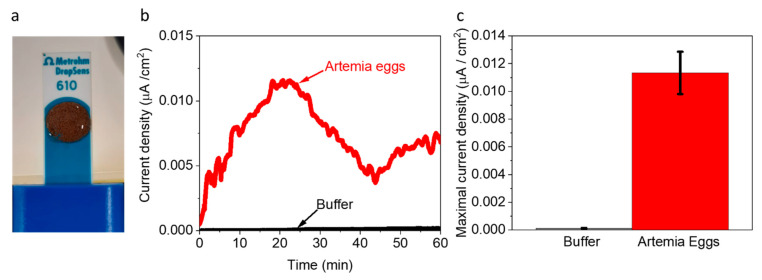
*Artemia* eggs produce an electric current in a BEC. Chronoamperometry measurements of *Artemia*’s eggs and the pure buffer under an applied potential of 0.5 V to the WE. (**a**) Picture of the measurement setup. The *Artemia* eggs (brown spots) entirely cover the surface of screen-printed electrodes. (**b**) Chronoamperometry measurements of the buffer (black) and *Artemia* eggs (red). (**c**) Maximal current densities of buffer (black) and *Artemia* eggs (red). The error bars represent the standard deviation over three independent repetitions.

Based on the insight that *Artemia*’s eggs release reducing molecules to their external solution, we wished to explore whether they can be used as electron donors to produce an electric current in a BEC configuration. To do this, 5 mg of *Artemia*’s eggs were placed on screen-printed electrodes made of (Methrohm Dropsens 610) graphite coated with Meldola’s blue as the working electrode (WE) (0.5 cm^2^), a graphite counter electrode 0.475 cm^2^, and Silver reference electrode (Figure 3a). The coating with Meldola blue was used since it displays electrocatalytic properties toward the oxidation of NADH [46]. Next, 5 mg of *Artemia* eggs solution was immersed on the screen-printed electrode, covering its entire surface area. Chronoamperometry measurements were conducted to measure the produced current over time under an applied potential of 0.5 V (sufficient to oxidize NADPH). As seen in Figure 3 and repeats in Figure S6, the *Artemia* eggs produce notable currents for more than 1 h. A maximal current of ~0.012 µA/cm^2^ was obtained, given that the whole area of the electrode was exposed to the external solution. This current density is about 40 times lower than previously obtained for intact cyanobacterial-based BECs [40]. We postulate that although the egg shells enable the release of redox-active molecules, this release is limited by its hard texture. Based on the 2D-fluorescence maps and electron mediation mechanisms that were previously reported for non-sea anemones organisms that habitat in marine aquatic environments [27,41], we suggest that *Artemia* eggs release redox active molecules that can donate electrons at the anode. Among these molecules is NADH. The fingerprint of amino acids obtained in the 2D-FM spectra indicates the release of peptides or proteins from the eggs. It is possible that some of these proteins are electroactive and may also contribute to the current production.

### 2.4. Nematostella Produce Electric Current in BEC

**Figure 4:**
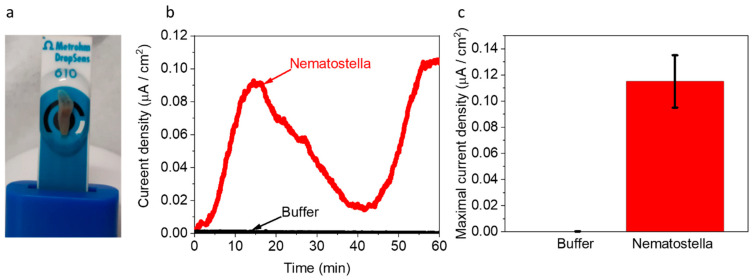
*Nematostella* produces an electric current in a BEC. Chronamperometry of *Nematostella* and pure buffer were measured for 1 h using screen-printed electrodes with an applied potential of 0.5 V on the WE. (**a**) A picture of the measurement setup. The *Nematostella* covers area of 0.31 cm^2^ (that is 60% of total WE area). A drop of 100 µL buffer covers *Nematostella* and the screen-printed electrodes and apply as an electrolyte. (**b**) Chronamperometry of buffer (black) and *Nematostella* (red). (**c**) Maximal current density of buffer (gray) and *Nematostella* (red). The error bars represent the standard deviation over three independent repetitions.

Next, we wished to explore whether *Nematostella* can produce electricity in a BEC. 1 unit of *Nematostella* was placed on screen-printed electrodes with (Methrohm Dropsens 610) (Figure 4a). The measurements were conducted at 18 °C. We postulated that a temperature change might have inflicted a small change in the electrochemical reactions and perhaps a bigger influence on the biological responses of the *Nematostella*. However, in this study, we wished to focus on optimal cultivation conditions that are non-stressful for the *Nematostella*. Such conditions may extend its viability and thus the duration of the current production. The contact area of the *Nematostella* and the anode was determined by image J software to be ~0.31 cm^2^ (~60% of its total area). This area was used for the calculation of the current density. As in the case of the *Artemia*’s eggs, the *Nematostella* produced current for more than 1 h, with a maximal current density of ~0.11 µA/cm^2^ (Figure 4c and Figure S7). Such current density is about 4 times lower than obtained for intact cyanobacterial-based cells [40]. Based on the 2D-FM spectra (Figure 1c,d), we suggest that NADH is released from the *Nematostella* and donates electrons at the anode to produce current. However, based on the absorption spectra of the external solution (Figure 1a,b), we conclude that many other biomolecules are released from the *Nematostella* and may consist of additional electron donors rather than NADH. The versatility of the current may originate from different secretion rates and composition of molecules by the organisms. Previous works in the field of bio-electrochemistry were mostly done on unicellular organisms. The higher complexity and multi-compartment structure (cells, tissue, etc.) of the sea anemones may result in divergent homeostasis that may lead to a bigger variation in the rate of electroactive species secreted by the organism. Direct current production originates from complex and highly dynamic biological reactions that probably cannot be stabilized into a constant current. Nevertheless, it is possible to couple the bio-electrochemical cell with an energy management system that will stabilize the power output.

A proposed mechanism for electron transfer in bio-electrochemical cells based on *Nematostella and Artemia’s* eggs.

**Figure 5:**
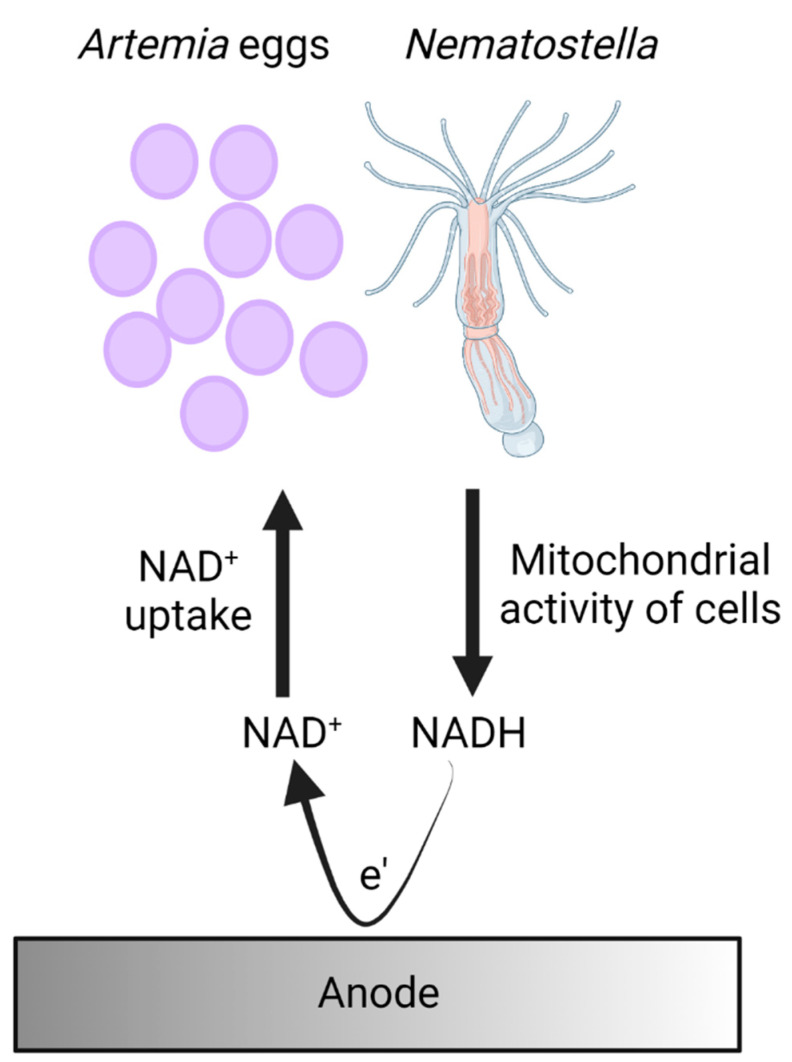
A proposed mechanism for electron transfer in bio-electrochemical cells based on *Nematostella and Artemia’s* eggs. Cells of *Nematostella and Artemia’s* eggs produce NADH by their mitochondrial activity. Upon association with the anode, some of the NADH molecules are released by the organisms and donate electrons at the anode to produce an electrical current.

Based on the identification of NADH in the external cellular matrix of *Nematostella* and *Atremia’s* eggs and previous mechanisms that were identified in other marine organisms such as cyanobacteria, microalgae, and seaweeds [27,28,29], we propose a model for the electron transfer in which the cells of the organisms produce NADH molecules by mitochondrial activity. Upon association with the anode, NADH molecules released from the organisms donate electrons at the anode to produce electrical current (Figure 5).

## 3. Materials and Methods

### 3.1. Materials

NADH ≥ 97% (HPLC) and Cyt C (From horse heart) were purchased from Merck, Darmstadt, Germany. *Artemia Salina* brine shrimp eggs were purchased from Ocean Nutrition, Essen, Belgium.

### 3.2. Cultivation of Nematostella Vectensis Culture

*Nematostella vectensis* were cultured in artificial seawater (Red Sea, Israel) at 12.5 ppt salinity in the dark at 18 °C, as previously described [47]. The *Artemia Salina* eggs that were used for measurements in this study were also used for the hatching of *Artemia Salina* that apply as food for *Nematostella*. The *Nematostella* were fed five days a week with freshly hatched *Artemia Salina* brine shrimps (Ocean Nutrition, Belgium).

### 3.3. Absorption and Fluorescence Measurements

The absorption measurements of the cells were done using Nanodrop 2000 UV-Vis spectrophotometer (Thermo Fisher Scientific, Waltham, MA, USA). The fluorescence measurements were done using a Fluorolog 3 fluorimeter (Horiba) with excitation and emission slits bands of 4 nm. Quantification of NAD(P)H concentrations was calculated based on the NADH calibration curve, which was based on increasing concentrations measured at (λ(ex) = 350 nm, λ(em) = 450 nm). The lines of diagonal spots that appear in all of the maps presented here and in the following figures result from the light scattering of the Xenon lamp [43].

### 3.4. Chronoamperometry Measurements

Chronoamperometry measurements were done using Plamsens3 potentiostat (Palmsens) connected to screen-printed electrodes (610, Metrohm Dropsens) with graphite coated with Meldola’s Blue working electrode, graphite counter electrode, and a silver reference electrode. The anode has a geometric surface area of 0.5 cm^2^. Meldola’s Blue coating is ideal for the determination of NADH at a low detection potential [46,48]. In the measurements of *Artemia* eggs, the entire area of the electrodes was covered by the eggs and 100 µL of pure cultivation buffer solution (of *Artemia* eggs). In the experiments of *Nematostella*, 1 *Nematostella* unit (76 ± 15 mg) is measured, covering an area of ~0.3 cm^2^. Evaluation of the coverage area was conducted by image analysis using Image J software. 150 µL of pure cultivation buffer solution (of *Nematostella*) was covering all electrodes and the *Nematostella*. The *Nematostella* taken for the experiments were starved from their *Artemia* food for ~24 h before the measurements to minimize the probability of the emission of digested residues. The current range of the Palmsense3 under potentiostatic mode can go down to 100 pA, which is sufficient for detecting our reported currents in the scale of 10’s of nA. The reason for the rather small current densities originates from the low biomass of Artemia eggs or 1 Nematostella unit.

### 3.5. Cyt C reduction Assay

1 *Nematostella* unit (76 ± 15 mg) or 5 mg *Artemia* eggs were incubated in their pure cultivation buffers for 1 h (This incubation time was chosen to enable correlating it with the time of the electrochemical measurements). The organisms were removed, and the external solutions were incubated for 1 h with 30 µM Cyt C. Absorption spectra of the oxidized Cyt C (without the external solution) and the mixtures were measured. The reduction degree of Cyt C was evaluated by the intensity ratio (λ = 550/540 nm).

## 4. Conclusions

To date, the science of BECs has been limited to microorganisms and photosynthetic macroorganisms. Here, we show for the first time that two representatives of the marine sea anemones: *Nematostella* and *Artemia* eggs can produce electricity in a bioelectrochemical cell configuration. We further demonstrate that *Nematostella* and *Artemia* eggs release reducing molecules into their external environment. We apply 2D-fluorescence measurements to show that the electron mediator NAD(P)H is among these molecules. This electron mediator was previously reported to be secreted by photosynthetic micro and macroorganisms [27,40,49]. As additional proof for the secretion of redox-active molecules, we show that *Nematostella* and *Artemia* eggs can reduce the electron acceptor Cyt C. This concept of sea anemones-based BECs may be a base for the development of future renewable energy technologies that will use the ability of different marine animals to produce clean energy.

## Supplementary Materials

The following supporting information can be downloaded at: https://www.mdpi.com/article/10.3390/ijms232315001/s1.

## Abbreviations

The following abbreviations are used in this manuscript:

MFC	Microbial fuel cells
BEC	Bio-electrochemical cells
BPEC	Bio-photo electrochemical cells
Cyt C	Cytochrome C
NAD(P)H	NADH or NADPH
