# Supplementary material for: Direct Electricity Production from Nematostella and Arthemia’s Eggs in a Bio-Electrochemical Cell
Source: Int J Mol Sci. 2022 Nov 30;23(23):15001. doi: 10.3390/ijms232315001 (PMC9738779; doi:10.3390/ijms232315001)
Supplement: Supplementary file 1 [file ijms-23-15001-s001.zip › ijms-2058980-supplementary.pdf]

## Supporting Information

# Direct Electricity Production from *Nematostella* and *Arthemia*'s Eggs in a Bio-Electrochemical Cell.

Yaniv Shlosberg*[1], Vera Brekhman[2], Tamar Lotan[2], and Lior Sepunaru*[1]

[1] *Department of Chemistry and Biochemistry, University of California at Santa Barbara, Santa Barbara, CA, 93106, United States*

[2] *Leon H. Charney School of Marine Sciences, University of Haifa, Haifa, Israel*

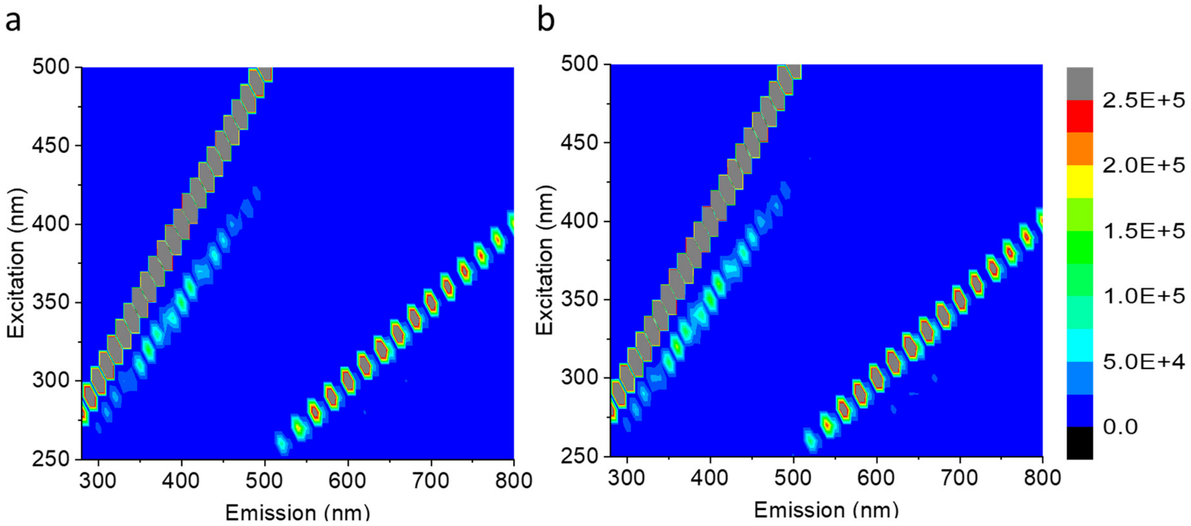


**Figure S1. 2D-Fluorescence maps of the pure cultivation buffers of *Artemia* eggs and *Nematostella*.** 2D-fluorescence spectra were measured for the pure buffers of Arthemia eggs and *Nematostella*, 2D-fluorescence spectra of the pure buffer of *Artemia* eggs **b** 2D-fluorescence spectra of the pure buffer of *Nematostella*. The lines of diagonal spots that appear in all of the maps presented here and in the following figures result from the light scattering of the Xenon lamp and Raman scattering of the water [39].

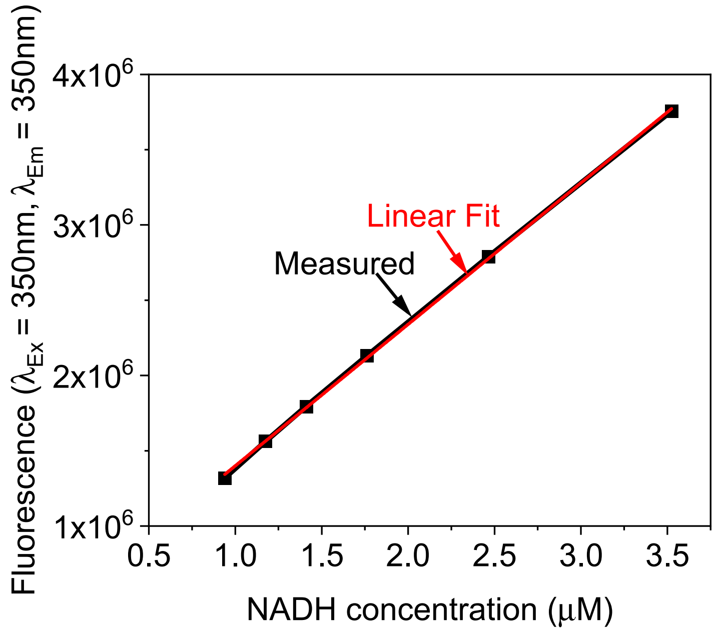


**Figure S2. NADH calibration curve. .** Fluorescence intensities was plotted against several NADH concentrations ($\lambda_{Ex}$ = 350, $\lambda_{Em}$ = 450 nm) (black). Linear fitting analysis (red).

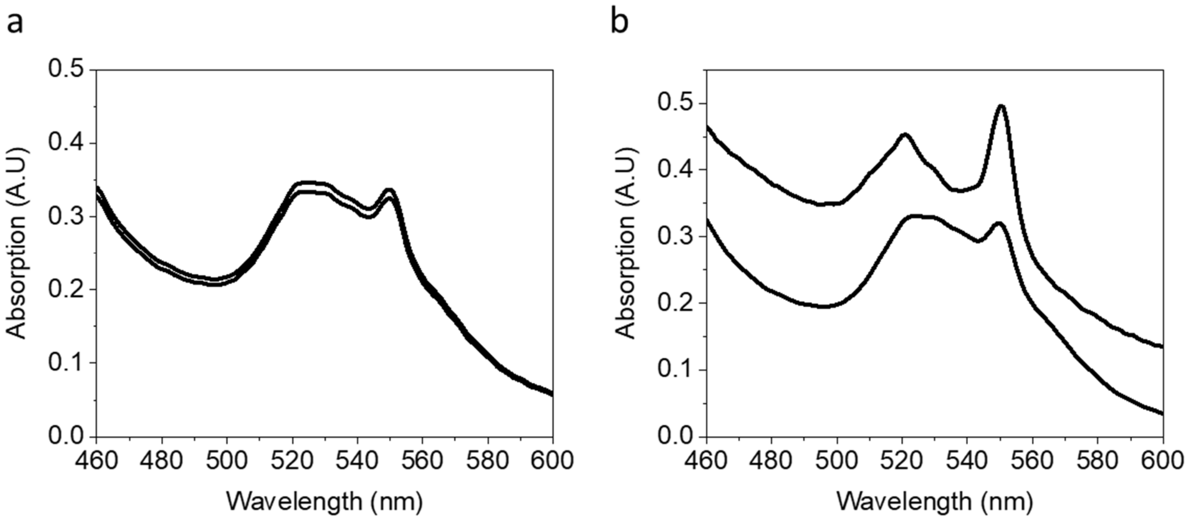


**Figure S3. Repetitions of Cyt C assay with the external solution of *Artemia* eggs and *Nematostella*.** **a** 2 additional repetitions of the experiment shown in Fig. 2a. The external solution of *Artemia* egg was measured after incubation with Cyt C (1 h). **b** 2 additional repetitions of the experiment shown in Fig. 2b The external solution of *Nematostella* was measured after incubation with Cyt C (1 h).

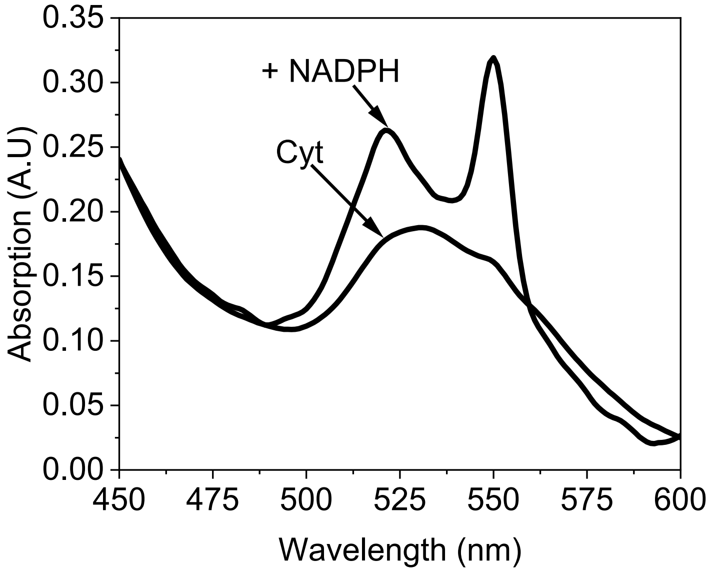


**Figure S4. NADPH reduce Cyt C.** 20 μM Cytochrome C was incubated for 1 h with 20 μM NADPH for 1h. Absorption spectra of pure Cytochrome C (black) and the mixture of Cytochrome C + NADPH.

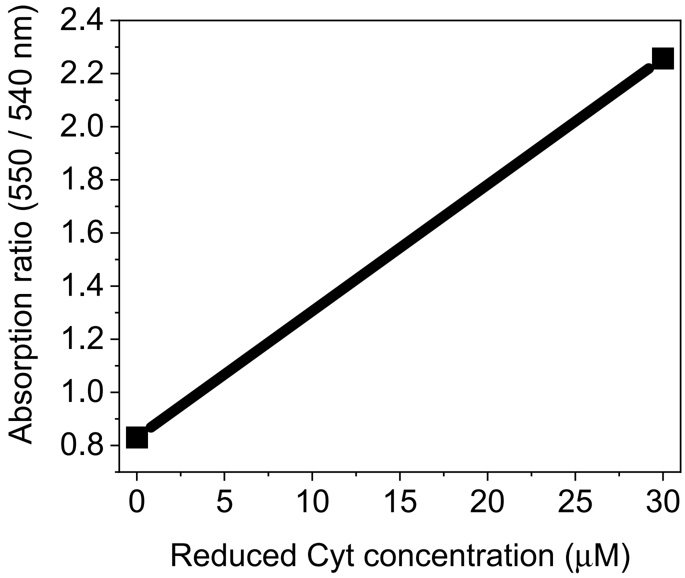


**Figure S5. A calibration curve of reduced Cyt concentration vs. absorption ratio (550 / 540 nm).** The calibration curve was plotted based on the absorption ratio (550 / 540 nm) of fully oxidized and fully reduce Cyt.

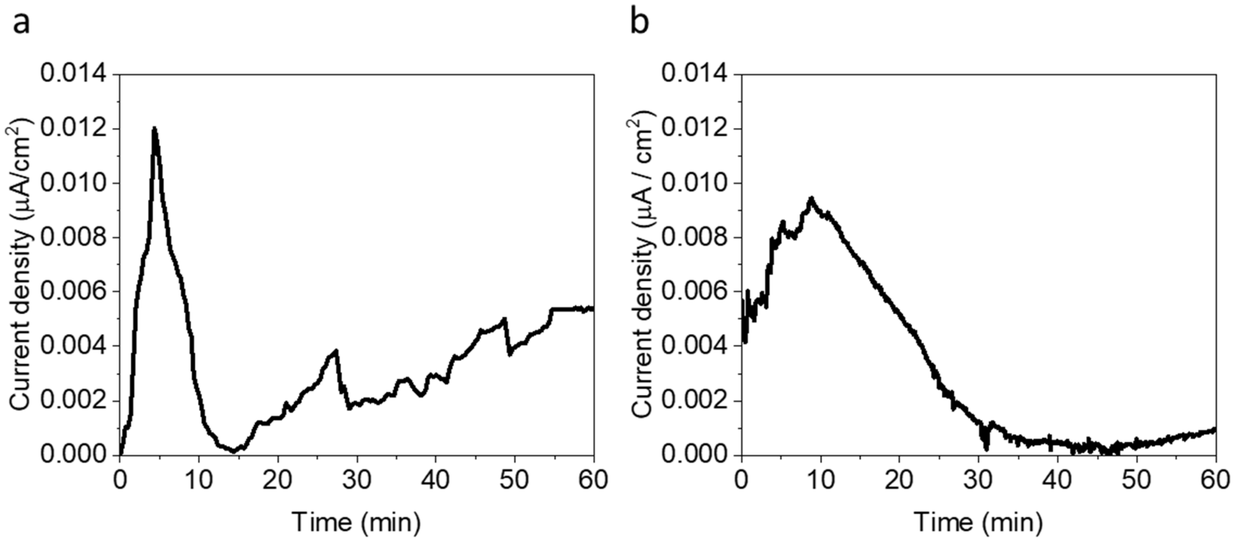


**Figure S6. Repetitions of the CA measurements of *Artemia* eggs.** Chronoamperometry measurements of *Artemia's* eggs and the pure buffer under an applied potential of 0.5 V to the WE**.** 2 additional repetitions of the experiment shown in Fig.3b. **a, b** CA of *Artemia* eggs was measured for 60 min.

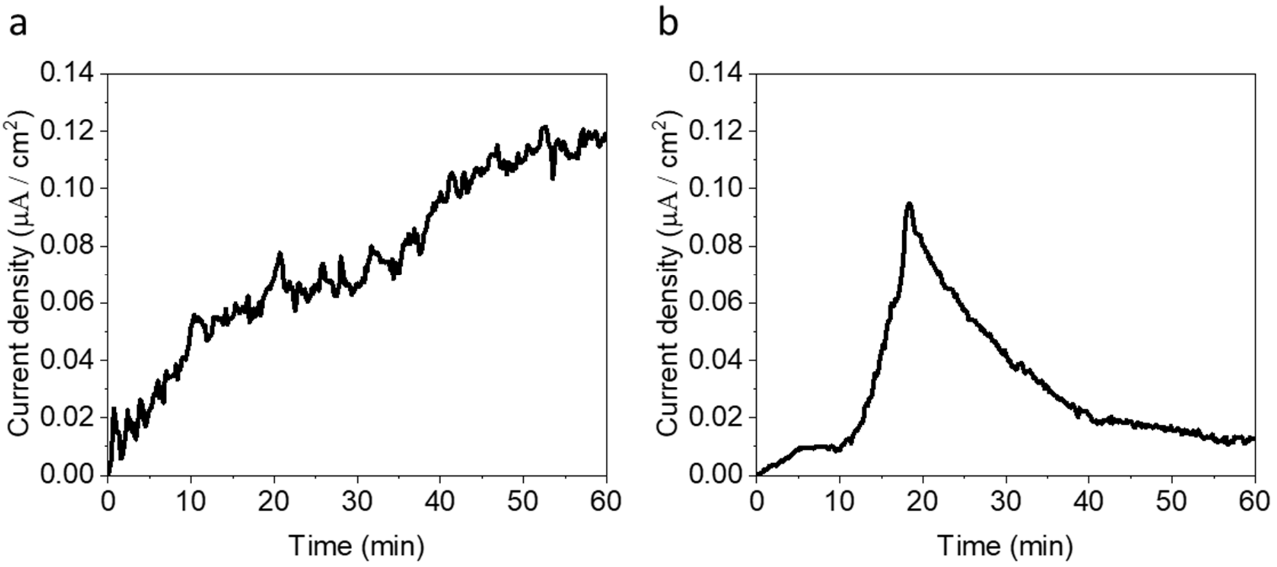


**Figure S7. Repetitions of the CA measurements of *Nematostella*.** Chronoamperometry measurements of *Nematostella* and the pure buffer under an applied potential of 0.5 V to the WE**.**2 additional repetitions of the experiment shown in Fig.4b. **a, b** CA of *Nematostella* was measured for 60 min.
